# Intentional Suppression Can Lead to a Reduction of Memory Strength: Behavioral and Electrophysiological Findings

**DOI:** 10.3389/fpsyg.2012.00401

**Published:** 2012-10-16

**Authors:** Gerd T. Waldhauser, Magnus Lindgren, Mikael Johansson

**Affiliations:** ^1^Department of Psychology, University of KonstanzKonstanz, Germany; ^2^Department of Psychology, Lund UniversityLund, Sweden

**Keywords:** inhibition, memory suppression, recognition memory, think/no-think, event-related potentials

## Abstract

Previous research has shown that the intentional suppression of unwanted memories can lead to forgetting in later memory tests. However, the mechanisms underlying this effect remain unclear. This study employed recognition memory testing and event-related potentials (ERPs) to investigate whether intentional suppression leads to the inhibition of memory representations at an item level. In a think/no-think experiment, participants were cued to either suppress (no-think condition) or retrieve (think condition) previously learned words, 18 or 0 times. Performance in a final recognition test was significantly reduced for repeatedly suppressed no-think items when compared to the baseline, zero-repetition condition. ERPs recorded during the suppression of no-think items were significantly more negative-going in a time window around 300 ms when compared to ERPs in the think condition. This reduction correlated with later recognition memory impairment. Furthermore, ERPs to no-think items from 225 to 450 ms were more negative-going in later phases of the experiment, suggesting a gradual reduction of memory strength with repeated suppression attempts. These effects were dissociable from correlates of recollection (500–600 ms) and inhibitory control (450–500 ms) that did not vary over the time-course of the experiment and appeared to be under strategic control. Our results give strong evidence that the no-think manipulation involves inhibition of memory representations at an item level.

## Introduction

Forgetting can be highly functional when being reminded of unpleasant or even traumatic autobiographical experiences. Studies employing the think/no-think paradigm show that it is possible to forget unwanted memories by repeatedly attempting to suppress them (Anderson and Green, 2001; Anderson et al., 2004; Anderson and Levy, 2009; Anderson and Huddleston, 2012; but see Bulevich et al., 2006; Waldhauser et al., 2011). In this experimental approach, subjects learn paired associates and are subsequently presented with one member of those stimulus pairs (cue) and instructed to either suppress (no-think condition) or retrieve (think condition) the other member of the pairs (target; Anderson and Green, 2001). In subsequent recall tests, memory for suppressed targets is often significantly diminished when compared to baseline items that have been learned, but not cued to be either suppressed or retrieved between study and test phase.

The suppression of unwanted memories correlates with the recruitment of inhibitory control areas in the prefrontal and parietal cortices, and reduced retrieval-related activity in the hippocampus and sensory processing areas (Anderson et al., 2004; Depue et al., 2007, 2010; Butler and James, 2010; Dieler et al., 2010). In event-related potentials (ERPs) studies, memory suppression is reflected in a series of negative amplitude peaks that predict later forgetting, a reduction of the recollection-related late parietal positivity, and a modulation of ERP slow-waves at anterior frontal electrodes (Bergström et al., 2007, 2009a,b; Hanslmayr et al., 2009; Mecklinger et al., 2009).

Based on these data, current theoretical accounts assume that the decrease of memory performance is due to inhibitory control mechanisms that act by dampening memory traces of no-think items (Anderson and Green, 2001; Levy and Anderson, 2002; Anderson and Levy, 2009). The present study put the inhibition-hypothesis to a direct test by combining the think/no-think procedure with an old/new item recognition test. Item recognition can be achieved on the basis of familiarity, which reflects the general memory strength of the target item (Ratcliff, 1978; Yonelinas, 2002). If intentional suppression in the think/no-think paradigm leads to an inhibition of the no-think target, this should result in reduced strength of the item representation, and should lead to decreased recognition performance for no-think compared to baseline items (cf. Hicks and Starns, 2004; Spitzer and Bäuml, 2007; Spitzer et al., 2009). However, such an effect has not yet been demonstrated (Tomlinson et al., 2009).

By measuring ERPs during the think/no-think phase we investigated whether the presumed inhibition of no-think items also leads to a reduction of neural signals of memory strength. Previous think/no-think studies showed that memory suppression affects the ERP correlates of conscious recollection, occurring around 500 ms after onset of a memory cue (Bergström et al., 2007, 2009a,b; Mecklinger et al., 2009). This suggests that conscious retrieval is under strategic control and can be intentionally avoided during suppression attempts. However, recollection can be independent of the strength of the actual memory trace (Bergström et al., 2009a). Thus, it remains to be shown that memory suppression reduces the neural signature of the memory representation, which would indicate the inhibition of the target memory trace (cf. Spitzer et al., 2009). In studies on recognition memory, dissociable ERP correlates of item memory strength typically occur between 300 and 500 ms at frontal electrode sites (Gonsalves et al., 2005; Rugg and Curran, 2007; Stenberg et al., 2009; Woroch and Gonsalves, 2010). Likewise, studies on cued recall report a modulation of ERPs with successful retrieval after 300 ms (Allan et al., 1996; Friedman and Johnson, 2000). Contrasting think and no-think trials, we expected ERPs related to memory strength to be more negative-going in the no-think condition. These analyses should also reveal the abovementioned correlates of inhibitory control and reduced recollection (Bergström et al., 2007, 2009a,b; Mecklinger et al., 2009). We expected a decrease of memory strength to be related to later memory impairment in the final item recognition test. Crucially, inhibition should gradually decrease the strength of the to-be-suppressed memory trace with repeated suppression, ultimately resulting in the inaccessibility of target representations. Such a gradual reduction is suggested by studies showing a linear decrease of recall performance in dependence of the number of suppression attempts (Anderson and Green, 2001; Hanslmayr et al., 2009). In the present study, a reduction of memory strength was expected to result in progressively more negative-going ERPs to no-think trials over frontal electrode sites in later parts of the experiment.

## Materials and Methods

### Participants

Twenty-four participants completed the whole course of the experiment. All participants had normal or corrected-to-normal vision, were native Swedish speakers and reported no history of neurological disease. Two participants had to be excluded due to recording errors. This resulted in a sample size of *n* = 22 (11 female) with a mean age of 27 years (range: 19–44). All subjects were right-handed as determined by a questionnaire (Chapman and Chapman, 1987, scores <19). In return for their participation, all participants received a lunch voucher worth SEK 90.

### Stimulus material

Three-hundred and sixty nouns were preselected as weakly related and emotionally neutral and were controlled for word length and frequency of occurrence in a comprehensive Swedish language corpus (Språkbanken, 2007). These words were subsequently combined into 180 weakly related word pairs, based on Latent Semantic Analysis (Landauer and Dumais, 1997). Pairs were split into six subsets consisting of 30 words each. The subsets were rotated across experimental conditions (baseline, think, no-think, new) across subjects. One-way ANOVAs comparing the controlled parameters between the six subsets showed them to be similar (all *F*s < 1, *ns*).

### Procedure

**Figure 1:**
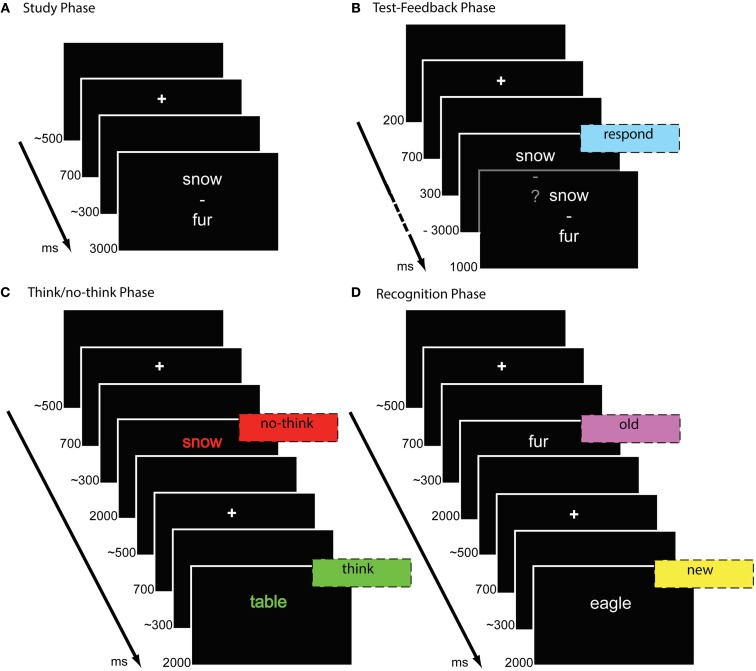
**Trial procedures in the different phases of the think/no-think experiment comprising study phase (A), test-feedback-cycles (B), think/no-think phase (C), and recognition test (D)**.

The experiment consisted of four phases: study, test-feedback, think/no-think, and final recognition phase. In the study phase (Figure 1A), participants were instructed to silently memorize each of the word pairs that were presented randomly in white on a black background for 3000 ms. The word pairs were presented in vertical fashion, with the cue words presented on the upper half of the screen and the target words presented on the lower half. Words were separated with a dash at the center of the screen. In the following test-feedback cycles (Figure 1B), the first words of the word pairs (cues) were presented for 3000 ms each. Words were printed in white color together with a question mark and words were selected in randomized order. Participants were instructed to verbally respond with the appropriate word previously presented at the lower position (target). Correct response terminated the presentation of the cue word and triggered the next trial. If the participant responded incorrectly or missed the response time-limit of 3000 ms, the correct response replaced the question mark for 1000 ms. After the cues of all word pairs had been shown, subjects received feedback in the form of their average total recall rate in the current cycle. Study and test-feedback phase were repeated up to three times or until the participant reached the criterion of a minimum of 66% correct responses. Response accuracy was recorded by key press from the experimenter.

In the following think/no-think phase (Figure 1C), participants were presented with 60 cue words at the center of the screen for 2000 ms each, 30 assigned to think and no-think conditions each. Each cue word was presented 18 times during the think/no-think phase. Thirty word pairs were assigned to the baseline condition and the cue words were not shown during the think/no-think phase. Before the phase started, participants were prepared that they are going to be presented with the first, upper words of the previously studied word pairs. When encountering a cue word in green color (think trials), participants were instructed to retrieve and think of the correct response but they should not verbalize the retrieved target. If participants could not remember the correct word, they were instructed to search memory for the correct word and try to retrieve the target every time they encounter the cue word in green color. For the no-think condition, instructions were as follows: “When a word is presented in red color, you should try to suppress the associated target word. Try to avoid thinking of the lower word of the word pairs! This word should never enter your consciousness! If you encounter a word in red color and you happen to think of the associated word, try to avoid thinking of the associated word the next time you encounter the red cue word.” The importance of these instructions was emphasized by a final instruction, stating “It is very important that you follow these instructions diligently! Green = Remember; Red = Suppress.” Subjects were discouraged from closing their eyes or removing their gaze from the stimulus. No overt responses were required during the think/no-think phase. The think/no-think phase included 1080 trials. The think/no-think phase was separated into three blocks. Each block consisted of 180 trials assigned to the no-think condition, and 180 assigned to the think condition. Each cue word was presented six times within each block. The trials within each block were presented in three types of clusters consisting of one to three trials of the same condition (think or no-think). Clusters consisting of think or no-think trials alternated. Each cue word was equally assigned to each of the three types of clusters under the constraint that the same word was never shown more than once within the same cluster.

Finally, memory for the target items was assessed in an old-new recognition test (Figure 1D). Participants were instructed to indicate whether an item was presented before (“old”), or had not been shown during the experiment so far (“new”), by pressing a corresponding button with the index finger of the left and right hand as long as the copy cue was presented. Mapping of the response types to left or right index fingers was counterbalanced across participants. All target words appeared in white color at the center of the screen for 2000 ms. Ninety trials comprised old items, i.e., target words of the critical word pairs, previously assigned to the no-think, think, or baseline condition. Another ninety trials consisted of new items that had not been presented previously during the experiment. Order of presentation was semi-randomized, with three new items and one item of each, baseline, think, and no-think condition being shown within six trials.

Each trial in all phases of the experiment was preceded by a fixation cross for 700 ms followed by a blank screen with a random duration of 200–400 ms. Trials were separated by a blank screen with a random duration of 400–600 ms. The different phases of the experiment and the blocks during the think/no-think phase were separated by breaks of variable length as self-paced by the participants. Each phase of the experiment was initiated by practice trials on five filler word pairs.

### Data analysis

#### Behavioral performance

To test the effects of the think/no-think manipulation on behavioral performance in the recognition test, we compared discrimination performance *Pr* (Hits – False Alarms) including items from baseline, think, and no-think conditions. Results are reported for all items that were initially presented, and a selection restricted to correctly recognized old items that had been successfully learned in the study phase (i.e., learned hits), as assessed in the last test-feedback cycle. There were no significant differences in the proportion of learned items between think (*M* = 0.671, SD = 0.111), no-think (*M* = 0.695, SD = 0.135), and baseline conditions (*M* = 0.689, SD = 0.108), *F*(2, 42) < 1, *ns*. In addition to *Pr* we compared RTs for learned hits from the baseline, think, and no-think conditions. We assumed that behavioral responses related to memory performance occurred between 300 and 1500 ms, treating higher and lower values as outliers and excluding them from statistical analysis. This lead to the exclusion of *M* = 0.5 (SD = 0.67, range: 0–2), *M* = 0.64 (SD = 0.79, Range: 0–2), and *M* = 0.59 (SD = 0.8, range: 0–3) outliers for the baseline, think, and no-think conditions, respectively. Number of outliers did not differ between conditions, *F*(2, 42) < 1, *ns*. Single RTs were log-transformed before submitting them to statistical analyses for approximating a normal distribution of the mean values (see Ratcliff, 1993, for discussion). Statistical comparisons of behavioral performance were calculated in one-way repeated-measures ANOVAs comprising the factor Condition (baseline, think, no-think). Main effects in the ANOVAs were followed up by planned, two-tailed comparisons between the conditions.

#### EEG recording and pre-processing

EEG was recorded from 30 Ag/AgCl scalp electrodes with a 500 Hz sampling rate and amplified from DC to 100 Hz on a Neuroscan (El Paso, TX, USA) NuAmps system, referenced to the left mastoid and re-referenced offline to averaged mastoids. Data were corrected for eye blinks and vertical eye movements using two additional channels assessing activity in the vertical and horizontal electrooculogram (EOG), applying a linear regression approach as implemented in Neuroscan Edit 4.3. EEG was recorded during all phases of the experiment. The ERP data reported here were taken from the think/no-think phase. ERPs were derived for items from think and no-think conditions that were successfully learned in the study phase. Continuous EEG was epoched from −200 to 2000 ms around onset of the cue word. All epochs containing activity exceeding ±75 μV at the scalp electrodes were excluded from further analyses. Offline filters (0.3–20 Hz bandpass, −3 dB, 12 dB/octave roll-off) were applied before further analyses in order to increase signal-to-noise ratio. EEG activity was averaged over the whole epochs and baseline-corrected using the 200-ms prestimulus interval.

#### ERP analysis

Throughout the analyses, we included the same topographical factors: anterior/posterior (frontal: F3, Fz, F4; frontocentral: FC3, FCz, FC4; central: C3, Cz, C4; centroparietal: CP3, CPz, CP4; parietal: P3, Pz, P4; occipital: O1, Oz, O2) and Hemisphere (left, midline, right) in each of the selected time windows. Topographical effects are only reported in case of interaction with factors covering the experimental manipulation of interest.

Time windows were selected based on previous findings, visual inspection of the data, continuous *t*-tests for each sampling point at all anterior/posterior electrode rows, and ERP peak detection at electrodes Fz and Pz. The first selected time window from 100 to 140 ms captured the N1 peak with a mean latency of 108 ms (SD = 19.16) at Pz that was followed by significant differences between conditions (no-think > think), mainly at posterior electrodes, from 108 to 148 ms (*p* < 0.05). The second and third time windows shared significant differences at frontal, frontocentral and central electrode rows (176–248 ms). However, topographical differences and previous research indicating overlapping ERP activity of differential functional significance between ∼150 and 300 ms justified the construction of two time windows (Bergström et al., 2007, 2009b; Mecklinger et al., 2009). A time window between 175 and 225 ms was indicated by significant differences between conditions (no-think < think) at parietal and centroparietal (172–222 ms) electrodes and included a parietal N2 peak (*M* = 212 ms, SD = 36.48 ms; Bergström et al., 2009b). The following time window (225–250 ms) included a frontal P2 (*M* = 232 ms, SD = 50.15 ms) that was accompanied by significant differences between conditions (no-think > think) at occipital electrodes (222–250 ms; Bergström et al., 2007; Mecklinger et al., 2009). Starting at frontal electrode sites (282–476 ms) and continuing from 326 to 574 ms at parietal and lasting until 590 ms at occipital electrodes, a stretch of significant no-think < think activity defined the next three time windows. A time window between 300 and 500 ms is typically constructed to capture the effect of memory strength (Rugg and Curran, 2007). However, previous think/no-think studies identified neural correlates of inhibitory control at a similar latency (Mecklinger et al., 2009). We observed the occurrence of two amplitude peaks with separable topographical distributions, suggesting neural activity that may distinctively correlate with memory strength and inhibitory control. The first peak occurred at *M* = 332 ms (SD = 50.15 ms) at Fz and had a frontal distribution. The second peak had a mean latency of 476 ms (SD = 40.97 ms) at Pz and was most prominent at centroparietal and parietal electrodes. In order to avoid an overlap in neural activity, we defined two narrow time windows, from 300 to 350 ms and from 450 to 500 ms. The following 500–600 ms time window was assumed to be related to conscious recollection and was the same as in previous think/no-think studies (Bergström et al., 2009b). A time window from 650 to 900 ms included significant differences between conditions (no-think > think) at frontal electrode sites (628–994 ms; Bergström et al., 2007; Mecklinger et al., 2009). Finally, a 1400–1800 ms time window captured several stretches of significant differences (no-think < think) that were most pronounced at centroparietal electrodes (1426–1500, 1522–1590, 1630–1722, and 1766–1804 ms).

For the selected time windows, ERPs were first submitted to 2 × 6 × 3 repeated-measures ANOVAs comprising the factors T/nt (think, no-think), and the topographical factors given above. Significant interaction effects of T/nt with topographical factors were followed up at electrode rows suggested by previous research on memory suppression and retrieval. Averages to the think condition comprised a mean of 321 (190–477) artifact free trials and grand averages to no-think trials had a mean of 336 (179–456) artifact free trials.

In order to identify ERP components that relate to later memory impairment, we correlated ERP mean amplitude differences (think – no-think) in the selected time windows with RT differences (no-think – baseline) at the 18 selected electrode sites. We chose RT differences for this analysis since they provides a fine-grained measure of cognitive processing. Accuracy measures may be less sensitive in such an analysis given the overall high hit rates. Furthermore, correlating RTs with ERPs allowed us to investigate how different memory processes affect memory performance for correctly retrieved items. This question has not been investigated previously and adds to the measures of item-specific forgetting effects reported below and the correlation analyses reported in previous studies (e.g., Bergström et al., 2009b). We calculated Spearmans rho (*r*_s_) as a non-parametric correlation statistic. Significance levels were determined by a non-parametric permutation procedure (5000 permutations) in each time window to correct for the 18 multiple comparisons, using a family wise alpha level of 0.05 (DeLong et al., 2011; Groppe et al., 2011). Positive *r*_s_ indicates more effective memory suppression by correlating with more memory impairment.

We also investigated the neural correlates of forgetting at an item level, by contrasting ERPs between remembered and forgotten items. First, we restricted our analysis to the no-think condition to identify time windows that contained neural activity related to the forgetting of suppressed target items. We submitted the data to a 2 × 6 × 3 ANOVA with the factor R/f (remembered, forgotten) and the same two topographical factors as in the previous analysis. We used the same time windows as in the think versus no-think analysis. In case of significant effects involving the factor R/f, we determined whether the ERP differences were specific to forgetting in the no-think condition or whether they signified neural processes that were common to forgetting, irrespective of condition. We compared think and no-think items that were later remembered or forgotten in a 2 × 2 × 6 × 3 ANOVA with the factors T/nt (think, no-think), R/f (remembered, forgotten), and the same two topographical factors as in the previous analysis. Main effects of R/f indicated forgetting-related neural activity that is independent of condition. Interaction effects involving the factors T/nt and R/f indicated condition-specific forgetting effects. Interaction effects involving the factor R/f were then followed up within the think condition in the same manner as the R/f analysis in the no-think condition. Due to the low number of misses, only 15 subjects having more than 14 trials per condition were included in this analysis, resulting in *M* = 42 (14–71) and *M* = 287 (127–401) for no-think items, and *M *= 34 (14-67) and *M* = 290 (138–443) for think items that were forgotten and remembered, respectively.

Finally, in order to reveal the ERP correlates of reduced memory strength, we compared the ERP averages to think and no-think items over the first, second, and third block of the think/no-think phase. We conducted an analysis in a time window from 225 to 450 ms, taking into account think and no-think items in a 2 × 3 × 6 × 3 repeated-measures ANOVA with the factors T/nt (think, no-think), Repetition (block-I, block-II, block-III), and the same topographical factors as in the previous analyses. Repetition effects were followed up separately for the think and no-think conditions in 3 × 6 × 3 repeated-measures ANOVAs comprising the factors Repetition (block-I, block-II, block-III) and the same two topographical factors as in the previous analyses. For the repetition analysis of no-think trials over the course of the think/no-think phase, averages comprised the following number of artifact free trials: block-I, *M* = 113 (54–154), block-II, *M* = 115 (62–150), block-III, *M* = 111 (59–152). For the think condition, values were as follows: block-I, *M* = 106 (54–159), block-II, *M* = 108 (57–157), block-III, *M* = 108 (61–161).

In all ERP analyses, Greenhouse–Geisser corrections were used where appropriate as indicated by significant Mauchly’s tests of sphericity, and corrected *p*-values are reported with uncorrected degrees of freedom.

## Results

### Behavioral data

Taking into account both learned and unlearned items, i.e., irrespective of whether the items have been successfully retrieved in the test-feedback phase or not, *Pr* differed significantly between conditions *F*(2, 42) = 4.236, *p* = 0.021. *Pr* scores were significantly lower for no-think items when compared to baseline, *p* = 0.008, and the think condition, *p* = 0.0495 (see Table 1). Restricting analyses to learned items only, revealed a different pattern. *Pr* was again significantly different between the conditions, *F*(2, 42) = 4.345, *p* = 0.019, with no-think items differing significantly from baseline (*p* = 0.005). However, the think condition did not differ from baseline or no-think. In fact, recognition performance for think items was numerically lower than baseline. However, covariance and correlation analyses revealed that this unexpected tendency for a performance decrease was independent of the no-think forgetting effect. Baseline-corrected forgetting in the think condition (*Pr* think – *Pr* baseline) did not correlate with forgetting in the no-think condition (*Pr* no-think – *Pr* baseline; *r*_s_ = 0.184, *p* = 0.414). Crucially, when controlling for baseline-corrected forgetting in the think condition in an analysis of covariance (ANCOVA), the difference in *Pr* between baseline and no-think items remained significant, *F*(1, 20) = 5.961, *p* = 0.024. Forgetting in the think condition did not interact with the effect of Condition (baseline versus no-think), *F*(1, 20) = 1.144, *p* = 0.298.

**Table 1:** **Behavioral Data: *M* (SD) for *Pr* for all studied items and those that have been correctly recalled during the test-feedback recall test, and RT for learned hits**.

	Baseline	Think	No-think
*Pr* all	0.798 (0.109)	0.787 (0.123)	0.745 (0.137)
*Pr* learned	0.836 (0.094)	0.798 (0.116)	0.771(0.136)
RT learned	800 (65)	791 (69)	826 (86)

Comparing log-transformed RTs to learned hits revealed a significant main effect of condition, *F*(2, 42) = 4.921, *p* = 0.012, with longer RTs to no-think hits when compared to baseline (*p* = 0.046) and think hits (*p* = 0.01). There was no significant difference when comparing RTs between think and baseline (see Table 1 for non-transformed RT values).

### Think versus no-think ERP effects

**Figure 2:**
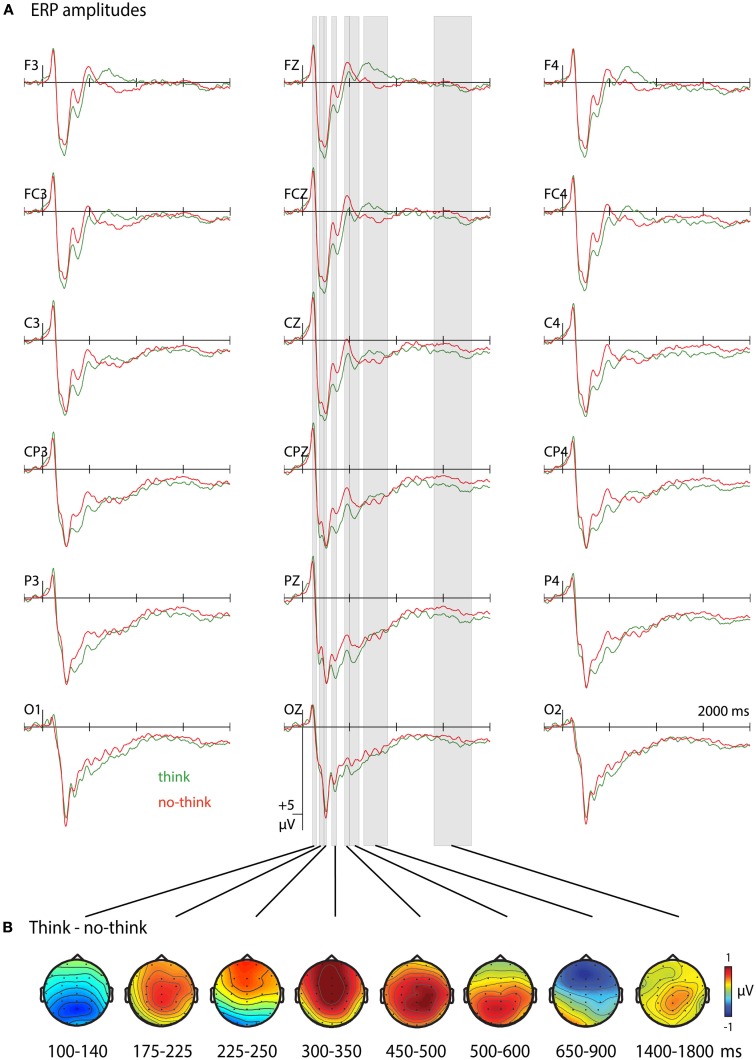
**ERP amplitudes during the think/no-think phase**. **(A)** Grand average ERPs to learned items from the think and no-think conditions. The waveforms are shown for the 18 electrode sites used for statistical analyses. Negative polarity is plotted upwards. **(B)** Topographical maps showing electrical activity over the scalp after subtracting ERPs during no-think trials from the one to think trials.

In the first time window (100–140 ms) ERPs to think trials showed more negative amplitudes, with a main effect of T/nt (see Table 2 for statistical values, Figure 2A for ERP waveforms and time windows, and Figure 2B for topographical maps). During the second time window (175–225 ms) more negative-going waveforms to no-think trials were observed that were most pronounced over centroparietal electrodes. More negative-going waveforms for the no-think conditions were also observed during the 225- to 250-ms time window. In this time window, waveforms to no-think trials were additionally more positive-going at occipital electrodes. This result pattern yielded a significant T/nt × Anterior/posterior interaction and significant main effects of T/nt at frontal, *F*(1, 21) = 6.569, *p* = 0.018, and occipital electrodes, *F*(1, 21) = 6.453, *p* = 0.019 (Bergström et al., 2007). In the 300- to 350-ms time window, a negative ERP higher in amplitude for no-think trials was obtained. This ERP was more pronounced over midline electrodes as suggested by the significant T/nt × Hemisphere interaction. Following up the significant T/nt × Anterior/posterior interaction effect during this time window revealed a main effect of T/nt at the frontal electrode row, *F*(1, 21) = 8.161, *p* = 0.009 (Rugg and Curran, 2007; Stenberg et al., 2009). Peaking between 450 and 500 ms at right centroparietal electrodes, another widespread negative-going ERP modulation was seen for no-think items. Between 500 and 600 ms we observed a T/nt × Anterior/posterior interaction, with more positive-going ERPs for think items over parietal electrodes, *F*(1, 21) = 5.37, *p* = 0.031 (Bergström et al., 2007, 2009a; Rugg and Curran, 2007; Mecklinger et al., 2009). In the 650- to 900-ms time window, we obtained a T/nt × Anterior/posterior interaction effect. Subsidiary analyses revealed a reliable positive-going ERP slow wave for the no-think condition at frontal electrodes, *F*(1, 21) = 19.361, *p* < 0.001 (Bergström et al., 2007; Mecklinger et al., 2009). Furthermore, the significant T/nt × Hemisphere and the T/nt × Anterior/posterior × Hemisphere interaction effects indicated that ERPs to think items were more positive over the left than the right hemisphere at posterior electrode rows. Finally, in the time window from 1400 to 1800 ms, more negative-going ERPs were observed for the no-think condition.

**Table 2:** **Results of the overall ANOVAs comparing ERPs to think versus no-think conditions in the selected time windows**.

Time window	T/nt		T/nt × Anterior/posterior		T/nt × Hemisphere		T/nt × Anterior/posterior × Hemisphere	
ms	*F*(1, 21)	*p*	*F*(5, 105)	*p*	*F*(2, 42)	*p*	*F*(10, 210)	*p*
100–140	14.915	0.001	3.3	*ns*	<1	*ns*	1.271	*ns*
175–225	8.032	0.01	4.244	0.04	2.501	*ns*	1.203	*ns*
225–250	<1	*ns*	12.756	<0.001	1.279	*ns*	<1	*ns*
300–350	6.142	0.022	8.22	0.003	3.462	0.041	2.141	*ns*
450–500	6.18	0.021	2.64	*ns*	<1	*ns*	<1	*ns*
500–600	3.386	*ns*	3.56	0.035	<1	*ns*	1.515	*ns*
650–900	2.027	*ns*	11.794	<0.001	3.294	0.047	2.890	0.022
1400–1800	4.927	0.038	1.243	*ns*	3.098	*ns*	2.335	*ns*

*α < 0.05; see Section “Think Versus No-think ERP Effects” and Figure 2 for direction of effects*.

### ERPs related to memory impairment

**Figure 3:**
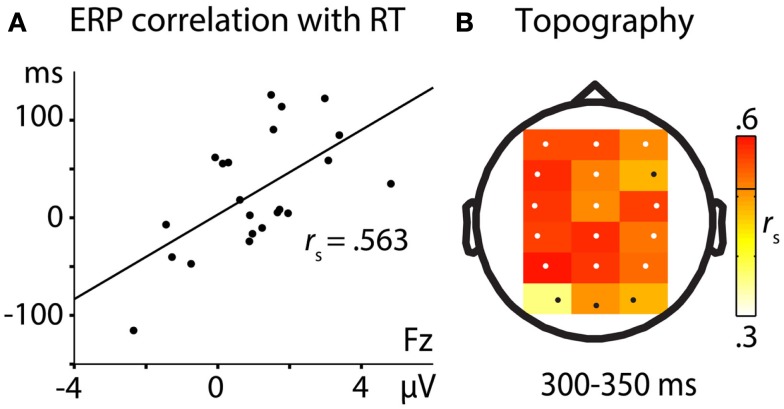
**Correlation of ERP differences (think – no-think) with RT differences (no-think – baseline)**. **(A)** Correlation at electrode Fz. The positive correlation is indicating more RT memory impairment with more negative no-think ERPs. **(B)** Topographical distribution of correlation effects at the 18 electrodes selected for statistical analysis. Significant electrodes (*p* < 0.05) are depicted in white. The threshold for significant correlation (*r*_s_ > 5.121) is depicted as a black line in the color bar.

More negative-going ERPs in the no-think condition in relation to ERPs to think trials predicted longer RTs for no-think when compared to baseline hits. The only significant correlational pattern of ERP amplitude differences with later memory impairment was obtained between 300 and 350 ms. Correlations were most pronounced across frontal, left hemispheric, and parietal electrodes (see Figure 3).

**Figure 4:**
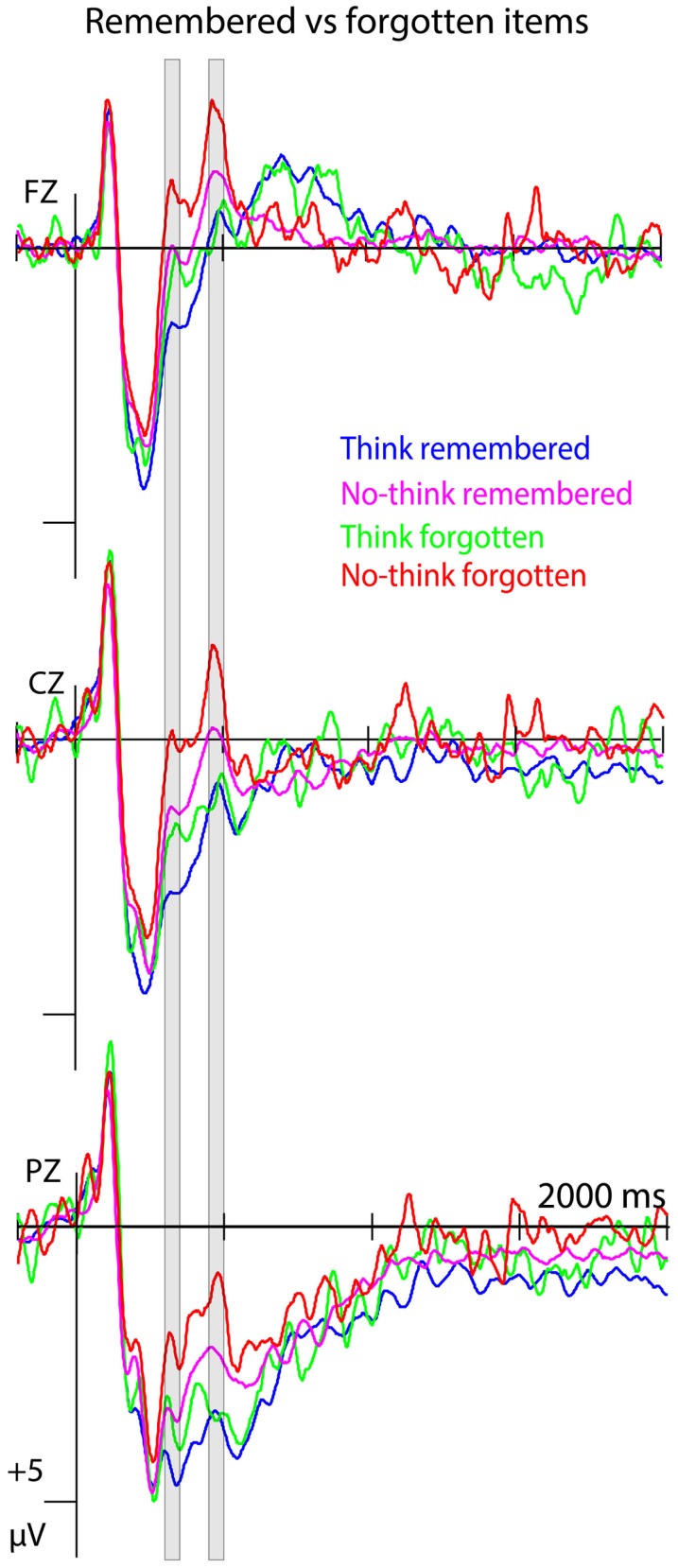
**Forgetting effects at the item level**. ERPs to forgotten and remembered items from both think and no-think conditions at midline electrode sites (*n* = 15). Negative polarity is plotted upwards.

Event-related potentials to forgotten no-think items differed from remembered no-think items in the 300- to 350-ms time window, with ERPs to forgotten items being more negative-going, as indicated by a main effect of R/f, *F*(1, 14) = 5.183, *p* = 0.039. Comparing ERPs to forgotten and remembered items between think and no-think conditions revealed a main effect of T/nt, *F*(1, 14) = 6.880, *p* = 0.02, and a main effect of R/f, *F*(1, 14) = 9.759, *p* = 0.007, but no interaction effects (*F*s < 1.511, *p*s > 0.238; see Figure 4).

In the 450- to 500-ms time window, there was a main effect of R/f in the no-think condition, *F*(1, 14) = 16.025, *p* = 0.001, with ERPs to forgotten items showing a clear negative peak. The ANOVA comparing ERPs to remembered and forgotten items in think and no-think conditions revealed a main effect of T/nt, *F*(1, 14) = 17.751, *p* = 0.001, and a trend for a T/nt × R/f interaction, *F*(1, 14) = 4.368, *p* = 0.055. To account for the lower power in this analysis, given the low signal-to-noise ratio and the small sample size, we followed up this non-significant trend. No main or interaction effects involving the factor R/f emerged in the think condition (*F*s < 1.589, *p*s > 0.193; see Figure 4).

### Repetition effects related to memory strength

Event-related potentials were sensitive to repetition effects from the 225- to 250-ms time window until 450 ms, with more negative-going ERPs for the no-think condition in later blocks of the think/no-think phase. The ANOVA comparing ERPs to think and no-think items across blocks of the think/no-think phase revealed a main effect of T/nt, *F*(2, 42) = 6.944, *p* = 0.015, a main effect of Repetition, *F*(2, 42) = 5.172, *p* = 0.01, and a T/nt × Hemisphere interaction, *F*(2, 42) = 3.873, *p* = 0.045. Crucially, we also obtained a significant T/nt × Repetition interaction, *F*(2, 42) = 4.405, *p* = 0.018, suggesting more negative-going ERPs in the no-think condition with later parts of the think/no-think phase.

**Figure 5:**
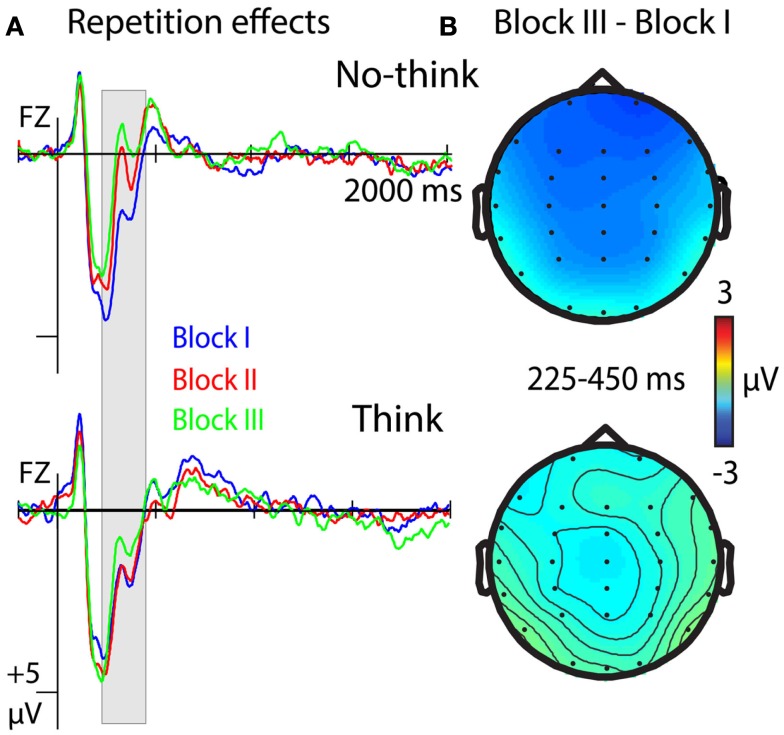
**ERP correlates of a reduction of memory strength with repeated suppression attempts for no-think items (top row) and think items (bottom row)**. **(A)** ERPs at electrode Fz from the three parts (block I, II, III) of the think/no-think phase. Negative polarity is plotted upwards. **(B)** Topographical difference between ERPs from the first (block-I) and the last part (block-III) of the think/no-think phase.

Statistical analysis for the no-think condition revealed a main effect of Repetition, *F*(2, 42) = 8.947, *p* = 0.001, and a significant Repetition × Anterior/posterior interaction, *F*(10, 210) = 6.904, *p* = 0.002 (see Figure 5A, top row). The effect of Repetition was largest at frontal electrodes (see Figure 5B, top row) where it followed a linear contrast *F*(1, 21) = 22.433, *p* < 0.001 (block-I > block-II > block-III). Testing for repetition effects in the think condition did not reveal any main, *F*(2, 42) = 2.097, *ns*, or interaction effects, *F*s < 1.557, *ns*, involving the factor Repetition (see Figure 5, bottom row).

## Discussion

The present study investigated the effect of memory suppression in the think/no-think paradigm on behavioral and electrophysiological correlates of memory strength. The repeated intentional suppression of unwanted memories led to memory impairment in an item recognition test and was reflected in more negative-going ERPs at frontal electrode sites. These findings are in line with inhibitory accounts of forgetting (Levy and Anderson, 2002; Anderson, 2005), suggesting that memory suppression can lead to the attenuation of memory trace strength at an item level. Novel methodological approaches, the employment of a recognition test, investigating memory impairment even for correctly recognized items by means of RT analysis, and showing repetition effects on ERP correlates of memory suppression, gave important insight on the different processes underlying the inhibition of unwanted memories.

To our knowledge, the present study is the first to show below-baseline suppression effects for no-think items in a recognition test. Memory suppression led to reduced discrimination performance for no-think items and this held even when constraining analyses to items that have been learned during study. Moreover, suppression not only affected accuracy measures but also led to longer reaction times for hits. Only two previous studies have investigated no-think effects on recognition memory. Marx et al. (2008) reported a no-think effect in a speeded recognition task. In that study, however, recognition performance for no-think items was compared with the think condition only and could merely indicate less gain for no-think items. Tomlinson et al. (2009) employed a forced-choice recognition test after the think/no-think phase and did not obtain suppression effects. This null effect may have resulted from the fact that recognition memory tests were preceded by recall tests and thus, any effects on recognition may have been counteracted by preceding retrieval attempts. Our finding corroborates the studies by Anderson and colleagues using independent probe tests (Anderson and Green, 2001; Anderson et al., 2004). These studies show that forgetting also occurs in response to recall cues that have a pre-experimental semantic relationship to the target items, but that have not been shown during the experiment (cf. Anderson and Green, 2001). Independent probe tests are considered to rule out that forgetting is due to a lowered effectiveness of the cue-target relationship in eliciting retrieval of a target. However, the study by Tomlinson et al. (2009) indicates that forgetting in independent probe recall tests can be due to interference. Engaging in a motor task upon cue presentation led to comparable forgetting in independent probe tests as intentional memory suppression (Tomlinson et al., 2009). Item recognition, in contrast, is dependent on the availability of the item memory trace alone, as trace strength is the only free variable in the matching computation between the study and test episodes (Ratcliff, 1978; Geiselman et al., 1983; Tulving, 1983; Bjork, 1989; Yonelinas, 2002). Thus, our results support the inhibition account and indicate that intentional suppression in the think/no-think paradigm results in the weakening of individual item memory traces, while circumventing the interpretational problems of independent probe tests (Hicks and Starns, 2004; Spitzer and Bäuml, 2007; Spitzer et al., 2009). As indicated by the no-think effects on RT, memory suppression does not only lead to forgetting, but also affects the retrieval of correctly recognized items.

Repeated retrieval practice for think items did not lead to an increase in recognition performance. For learned items, recognition accuracy was even numerically decreased in the think condition when compared to baseline. Interpretations of this pattern have to remain speculative at this point and await further studies that specifically investigate the effect of repeated retrieval practice on highly overlearned memories. It is possible that the excessive retrieval practice of the association between the cue and the target in the think/no-think phase rendered the test probe in the recognition test ineffective in eliciting a correct response for the single target item (Tulving and Thomson, 1973; Buchler et al., 2008). In line with our findings, memory performance is often not enhanced in independent probe tests that are designed to test memory strength of the target items alone (Anderson and Green, 2001; Anderson et al., 2004; Bergström et al., 2009b; Anderson and Huddleston, 2012). Importantly, as shown by correlation and covariance analyses, behavioral performance in the think condition was independent of forgetting in the no-think condition. The ERP data give further evidence for this independence of effects, since ERPs between think and no-think conditions differed substantially, and these differences predicted later memory impairment.

Memory suppression in the no-think condition led to more negative-going ERPs for the no-think condition over frontal scalp areas. We have strong reasons to assume that this effect reflects a reduction of ERP signals of memory strength. In the comparison of think and no-think conditions, the difference emerged in a time window around 300 ms. This matches studies on recognition memory that show that more positive-going ERPs (attenuated negativity) at frontal electrode sites in a comparable time window are associated with familiarity-based recognition judgments (Vilberg et al., 2006; Rugg and Curran, 2007; Stenberg et al., 2009). In line with this, more negative ERPs in the 300- to 350-ms time window indicate reduced recognition performance, as shown in our analysis investigating item-specific forgetting effects. While this naturally holds true for all items, irrespective of whether forgetting is due to memory suppression or not, the no-think condition entails a stronger reduction of memory strength. The ERP difference between think and no-think conditions correlated with later memory impairment between subjects and highest correlations were obtained at left frontal and parietal electrode sites at which neural activity is known to reflect memory strength (Gonsalves et al., 2005; Rugg and Curran, 2007; Stenberg et al., 2009). The spatiotemporal characteristics of this correlation pattern makes sense when assuming that the less positive-going ERPs during the think/no-think phase reflect the same process as the one that underlies reduced recognition performance (cf. Yonelinas, 2002). In addition to the item-specific effects, this result pattern indicates that a modulation of memory strength does not only relate to forgetting, but also correlates with reduced memory performance for correctly recognized items. Finally, ERPs between 225 and 450 ms at frontal electrode sites were more negative-going in later phases of the experiment, resembling the linear reduction in memory performance with increasing number of no-think trials observed in previous studies (Anderson and Green, 2001; Depue et al., 2006; Hanslmayr et al., 2009; Anderson and Levy, 2010). The behavioral reduction in recognition performance, the electrophysiological patterns, and the correlation between these measures strongly suggest that the no-think condition entails a gradual reduction of memory strength with repeated suppression attempts.

Replicating and extending previous studies, we observed ERP differences between think and no-think conditions in several time windows. We found an N2-like negativity between 450 and 500 ms. Differences in this time window predicted forgetting at an item level exclusively for the no-think condition. This indicates that the ERP peak reflects processes that are related to the active suppression of memory traces, corroborating the assumption that it signifies the recruitment of inhibitory control mechanism (Bergström et al., 2009a,b; Mecklinger et al., 2009). We also found indicators of reduced recollection for no-think items, emerging in less positive-going ERPs over parietal electrode sites between 500 and 600 ms (Bergström et al., 2007, 2009a; Mecklinger et al., 2009). ERP activity in this time window did not differentiate between remembered and forgotten items. This is in line with theories on recognition memory, stating that recollection is not a necessary process for item recognition (cf. Yonelinas, 2002). Contrasting our finding with the 300- to 350-ms effect, forgetting is observed irrespective of whether recollection was successfully avoided during the think/no-think phase or not. As in previous studies, neither the recollection-related effects, nor the inhibitory control component correlated with individual differences in memory impairment (Bergström et al., 2009a,b; Mecklinger et al., 2009). Furthermore, ERPs observed in these time windows did not vary with repeated suppression attempts. This implies that the ERPs observed in these time windows reflect processes that can be strategically employed, irrespective of actual memory strength of the to-be-suppressed target (Herron and Wilding, 2005; Cabeza et al., 2008; Bergström et al., 2009a; Mecklinger, 2010).

We found a frontal, positive-going slow wave for no-think items in the 650- to 900-ms time window (Bergström et al., 2007; Mecklinger et al., 2009). As reviewed by Mecklinger (2010), this slow wave may reflect processes acting in regulating the accessibility of unwanted memories, even if it does not directly correlate with memory suppression (Mecklinger et al., 2009). We also obtained a negative-going slow wave for no-think items in a late time window from 1400 to 1800 ms. This fits the results in a study by Hanslmayr et al. (2009), reporting sustained negative ERPs for no-think items that possibly indicate the strategic control and monitoring of memory retrieval. In addition to these late ERP effects, we found several early components to differ between think and no-think conditions. We observed more negative ERPs to no-think items between 175 and 225 ms. Corresponding to our results, Bergström et al. (2009b) found an early negativity for no-think items between 175 and 225 ms that was exclusively observed in participants that applied a direct suppression strategy to avoid thinking of the target, without retrieving substitutive memories. In contrast to Bergström et al. (2009b) we did not obtain a relationship of this ERP effect with later memory impairment. It is likely that this lack of correlation is due to the fact that we did not explicitly instruct subjects to use a direct suppression strategy. A relationship with a direct suppression strategy was confirmed in a *post hoc* correlation analysis with a post-experimental self-report questionnaire (Hertel and Calcaterra, 2005), assessing how often participants used a direct suppression strategy versus focusing on substitutive representations. We found that ERP differences between think and no-think conditions correlated with a direct suppression strategy at electrodes F3 and FC3 (*r*_s_ > 0.563, *p* < 0.05). Early differences between think and no-think conditions during the 100- to 140-ms and 225–250 ms time windows likely reflect higher attention to cues of the think condition, as extensively discussed by Bergström et al., 2007; cf. Mecklinger et al., 2009). In contrast to previous studies, these differences occurred earlier in the present data, already with an enhanced N1 peak for think items. The N1 often occurs around 100 ms, is thought to reflect top-down attentional control on early visual processing areas (e.g., Vogel and Luck, 2000), and has been shown to be sensitive to task instructions (Potts et al., 2004). The 225- to 250-ms pattern resembles an effect observed in previous think/no-think studies between 200 and 300 ms, and likely resembles a frontal selection positivity/posterior selection negativity to think items as discussed by Bergström et al. (2007). As indicated by the repetition analysis, the effect in the 225- to 250-ms time window overlapped with a reduction of memory strength for no-think items.

Finding several ERP components that differentiated between think and no-think conditions suggests that widespread control networks are involved in the successful suppression of unwanted memories (Depue, 2012). In order to achieve the identification of several ERP components and to distinguish them in their functional significance, we selected time windows based on previous research and inspection of the current dataset. The analysis strategy chosen for the present study conforms with a common statistical approach to ERP data (Picton et al., 2000; Dien and Santuzzi, 2005; Luck, 2005). However, selecting nine different time windows and statistical testing for each of these time windows, entails that some of the identified obtained statistical significances could in fact be false-positives (cf. Luck, 2005). Our ERP effects are to a large extent backed up by previous findings and the different ERP components appear to have distinguishable functional roles in memory suppression. Nonetheless, studies that selectively investigate some of the components described in the present study and that employ statistical methods that take care of multiple comparison problems are necessary to confirm our conclusions (cf. Bergström et al., 2009a,b).

Our results have important implications for the interpretation of memory suppression effects in the think/no-think paradigm. Most importantly, the observation of reduced recognition memory performance for no-think items, together with frontal phase-sensitive ERP modulation indicate that the no-think instruction can affect the memory strength of suppression targets at an item level. This strongly suggests that forgetting effects in think/no-think studies can be due to inhibition. Our measures of memory strength are robust against alternative explanations for no-think effects (Tomlinson et al., 2009). Neither performance in item recognition tests (Ratcliff et al., 1990), nor early, frontal ERP effects are sensitive to interference (Norman et al., 2008). It is possible that episodic interference, as pointed out by Tomlinson et al. (2009), can affect performance in recall tests that depend on conscious recollection and that are more susceptible to interference (Tulving, 1983; Bjork, 1989; Koriat and Goldsmith, 1996). Importantly, our data suggest that even if this can be the case, forgetting due to reduced memory strength is independent of a reduction of recollection. The attenuated late, parietal ERP effect indicating reduced recollection was not modulated by repeated suppression attempts, whereas the earlier, frontal positivity diminished over the time-course of the experiment. Also, early ERP differences correlated with memory impairment, at an item level and for retrieval of correctly recognized items, whereas ERPs in the recollection-related time window did not, further indicating that ERP reductions in this early time window tap into the memory processes that are important for a reduction in item strength. Our results add to the literature on the ERP correlates of cued recall. Studies in this field report more positive-going ERPs for successfully recalled items after 300 ms (Allan et al., 1996; Friedman and Johnson, 2000). We show that intentional suppression can affect these early correlates of recall for no-think items, but suggest that they are constituted by modulations of item memory strength that dissociate from strategically controlled recollection.

Previous neuroimaging studies on the think/no-think effect did not show such a dissociation. FMRI studies are sometimes not able to clearly dissociate between the subregions of the MTL involved in recollective and strength-based remembering (Kirwan et al., 2007). Thus, decreased MTL activity observed in fMRI studies employing the think/no-think paradigm could reflect both, reduced recollection as well as reduced memory strength (Anderson et al., 2004; Depue et al., 2007). As pointed out above, it is crucial to show a selective reduction of memory strength to conclude that the no-think effect involves inhibition, since a reduction in hippocampus-based recollection can be due to strategic avoidance and/or interference, but not necessarily to an effective attenuation of item memory traces (Norman et al., 2008; Norman, 2010). Previous ERP studies can be interpreted in the light of the present findings. In particular, Bergström et al. (2009b) showed that ERPs between 300 and 500 ms were more negative-going for no-think items in the direct suppression group, but not in participants that retrieved substitutive memories in order to avoid thinking of the target. Crucially, only the direct suppression group showed inhibitory forgetting on independent probe test. The authors interpreted the ERP difference for the direct suppression group as reflecting inhibitory control mechanisms (Bergström et al., 2009b). This is in line with previous studies, directly relating ERP negativities to inhibitory control (Mecklinger et al., 2009). However, it is as feasible to assume that negative ERPs can reflect a reduction of memory strength. Importantly, our findings indicate that ERP correlates of inhibitory control and memory strength may overlap, but differ with respect to sensitivity to repetition. Whereas frontal ERPs related to memory strength are reduced over the time-course of the experiment, centroparietal correlates of inhibitory control are unaffected by repetition and appear to be under strategic control (Bergström et al., 2009a; Mecklinger et al., 2009).

In sum, our data provide a cornerstone for the assumption that intentional suppression in the think/no-think paradigm involves the inhibition of unwanted memories. We show that the strength of memory traces is reduced on measures that are robust against interference as an alternative explanation for forgetting effects. Our results help to dissociate between different cognitive processes related to the suppression of unwanted memories. The activation of executive control networks contributes to the memory impairment of unwanted memories. Forgetting then can result from an inhibition of memory traces at an item level, as reflecting in recognition memory impairment and decreased physiological signals of memory strength.

## Conflict of Interest Statement

The authors declare that the research was conducted in the absence of any commercial or financial relationships that could be construed as a potential conflict of interest.
